# Assessing Seasonality Variation with Harmonic Regression: Accommodations for Sharp Peaks

**DOI:** 10.3390/ijerph17041318

**Published:** 2020-02-18

**Authors:** Kavitha Ramanathan, Mani Thenmozhi, Sebastian George, Shalini Anandan, Balaji Veeraraghavan, Elena N. Naumova, Lakshmanan Jeyaseelan

**Affiliations:** 1 Department of Biostatistics, Christian Medical College, Vellore 632002, India; kavitha.opm@gmail.com (K.R.); mani.thenmozhi@gmail.com (M.T.); 2 Department of Statistics, St. Thomas College, Palai, Kerala 686575, India; sthottom@gmail.com; 3 Department of Clinical Microbiology, Christian Medical College, Vellore 632004, India; shalinianandan@cmcvellore.ac.in (S.A.); vbalaji@cmcvellore.ac.in (B.V.); 4 Friedman School of Nutrition Science and Policy, Tufts University, Boston, MA 02111, USA; elena.naumova@tufts.edu; 5 Department of Gastrointestinal Sciences, Christian Medical College, Vellore 632004, India

**Keywords:** time series, harmonic regression, seasonality, infectious disease, ARIMA/SARIMA, trends

## Abstract

The use of the harmonic regression model is well accepted in the epidemiological and biostatistical communities as a standard procedure to examine seasonal patterns in disease occurrence. While these models may provide good fit to periodic patterns with relatively symmetric rises and falls, for some diseases the incidence fluctuates in a more complex manner. We propose a two-step harmonic regression approach to improve the model fit for data exhibiting sharp seasonal peaks. To capture such specific behavior, we first build a basic model and estimate the seasonal peak. At the second step, we apply an extended model using sine and cosine transform functions. These newly proposed functions mimic a quadratic term in the harmonic regression models and thus allow us to better fit the seasonal spikes. We illustrate the proposed method using actual and simulated data and recommend the new approach to assess seasonality in a broad spectrum of diseases manifesting sharp seasonal peaks.

## 1. Introduction

Understanding temporal changes in disease occurrence in human populations is one of priorities in epidemiology, public health, and life science related disciplines. This lofty goal implies ability to describe, quantify, and examine temporal patterns, which include increasing or declining trends, seasonal patterns, unusual spikes associated with outbreaks or disappearance of periodic episodes that mark disease eradication. These temporal characteristics are often explored in order to detect emerging trends in populations of concern, determine the success of intervention programs on a population level, and to forecast the future temporal behaviors [1,2,3]. The temporal analyses are also performed to better understand the effects of contributing factors to changes over time.

It is well known that majority of infections exhibit strong seasonal patterns in disease incidence or prevalence [3,4,5,6,7,8,9,10,11]. Our own work illustrate that infections caused by bacteria, like *Vibrio* cholerae [4] and *Salmonella* [6]; by protozoa, like *Giardia* and *Cryptosporidium* [12]; and viruses, like Influenza [13,14] and Rotavirus [15,16] have well pronounced seasonal patterns, specific for the location, pathogenic strains, and the size and socio-demographic composition of the affected populations. 

We define ‘seasonality’ as systematic, repetitive, periodic fluctuations in disease incidence over the course of one year. Disease seasonality is characterized by the magnitude, timing, and duration of a seasonal increase [17]. We define the time of a seasonal peak, a parameter of interest, as the position of the maximum point on a seasonal curve. The maximum and minimum values on the seasonal curve, the difference between them, and the ratio of these values are the magnitude-related measures. These characteristics along with their uncertainty measures allow us to compare seasonal patterns across diseases, locations and populations and offer statistical inferences. The use of the δ-method, which can be applied to estimate the uncertainty measures within a framework of a harmonic regression model, simplified substantially the modeling and estimation procedures [17]. 

Disease occurrence is typically measured as a rate, based on the number of events per unit of time normalized for population of interest. When the population of interest is relatively stable, disease occurrence can be measured as counts observed in a particular location and time, generally known as ‘aggregated information’, which will not have denominator. In order to describe and examine temporal changes, counts of disease episodes need to be organized as a sequence which form a time series of events in a given population over a pre-specified time period with a pre-specified level of temporal resolution, such as daily, weekly, or monthly time series.

The compiled time series data are usually analyzed using two approaches [18]. One is ‘time domain approach’, which treats the time series of events as a function of time with the primary goal to explore a trend (rising or declining) and if so, to fit a forecasting model. The time domain approach may be thought of as regression of the present on the past. The other one is a ‘frequency domain approach’ which is based on the assumption that the behavior of a time series is likely to be decomposed using periodic functions. The focus of this approach is to determine the periodic components embedded in the time series. The frequency domain approach may be considered as regression of the present, which are wave like periodic patterns of peaks/dips can be modeled using sine and cosine functions [18]. The choice between the frequency domain and the time domain depends is essentially objective based [19]. Generally, Auto Regressive Integrated Moving Average (ARIMA) and Seasonal Auto Regressive Integrated Moving Average (SARIMA) methodologies are used for the time domain approach and regression methodologies are used for the frequency domain approach. Recently we proposed a method that use both approaches in a combined manner [18].

Regression models adapted for time series of counts are gaining popularity and broad acceptance [9,20,21,22,23]. To describe seasonal oscillations in a time series of counts, a parametric or non-parametric Poisson regression model is most commonly applied. A parametric Poisson model typically includes terms based on trigonometric sine and cosine functions, and often has been referred as harmonic regression models [5,6,7,8,10,24]. The standard sine and cosine functions are smooth and symmetric and thus are appropriate for diseases that exhibited steady seasonal rise and decline. The use of well-defined function allows for clear and transparent comparison and interpretation of results. However, the use of Poisson-based approach should be taken with caution, because the actual data rarely satisfy the assumption of mean-dispersion equality needed for a Poisson distribution. Generalized Poisson and Negative Binomial models account for under and over dispersed count data; yet, these models can fit well only data with a moderate level skewness. When a disease of interest shows the sharp peaks and prolong periods of low incidence, the traditional harmonic Poisson or Negative Binomial regression models might still underestimate peaks and overestimate the dips. Therefore there is a need to adapt the existing models with parameters that would capture the sharp peak. 

This communication aims to improve the model to capture the specific behavior with characteristic sharp peaks and prolong periods of low disease incidence. We propose a two-step procedure, when at the first step we build a basic model, which allow to estimate the seasonal peak timing. At the second step we apply an extended model based on newly proposed sine and cosine transform functions, which mimic a quadratic term and thus better fit the seasonal spikes. We illustrate the method using actual and simulated data. As the motivational examples, we selected cases of hospitalizations due to *Salmonella* infections among older adults (those aged 65 years and older) in the U.S. during 1991–2002 [2], laboratory confirmed cases of *Shigella* infections among patients coming to the emergency department of the Christian Medical College and Hospital, in Vellore, India over a decade, and monthly pneumonia and influenza death counts in the U.S. for 11 years, from 1968 to 1978 [18]. We also used simulated data with predefined trend and seasonal pattern to illustrate the proposed two-step approach. 

## 2. Methods

### 2.1. The Base Model 

The conceptual framework to describe periodic oscillations is expressed as

Z_t_ = µ + γ cos(2πωt + φ) + ε_t_ (1)

where, Z_t_ is a time series of an outcome of interests measured at time t, t = 1, 2,...., N with N—An effective length of a time series (number of observations); µ is the constant reflecting the general baseline of Z_t_; the periodic component has a frequency of ω, an amplitude of γ, and a phase angle of φ; and ε_t_, are independently and identically distributed normal random variables with E[ε_t_] = 0 and Var[ε_t_] = σ^2^. This model describes seasonal behavior by a cosine function with symmetric rise and fall over a period of a full year. The locations of two points, the seasonal curve peak and nadir (lowest point), can be determined using a shift, or phase angle parameter, which reflects the timing of the peak relative to the origin. The shift parameter is expressed in the time units of a time series and can be used for seasonality comparison. The amplitude of fluctuations between two extreme points is controlled via parameter γ. If γ = 0, there is no seasonal increase. 

We assume that a period of oscillation or a cycle is known; thus, the frequency, the reciprocal of the period in t units, is a fixed number. Therefore, the model has three parameters—the constant, amplitude and phase. To ease the estimation, the model can be re-formulated as [17]

Z_t _ = µ + γ cos(2πωt + φ) + ε_t_ = µ + β_S_ sin(2πωt) + β_C_ cos(2πωt) + ε_t_ (2)

where, β_S_ = −γ sin φ and β_C_ = γ cos φ, the model parameters or beta coefficients. The temporal resolution of actual data can be reflected by ω = 1/M, where M depends on the unit of analysis, and is 4 for quarterly data, 12 for monthly data, 52.25 for weekly data, and 365.25 for daily data. A general framework of a regression model is sufficient to estimate the model parameters µ, β_S_, and β_C_. Furthermore, by using the δ-method, the estimates of peak timing and amplitude can be supplemented by the uncertainty measures [17].

This simple harmonic regression model can be applied to variety of scenarios and satisfy various forms of actual data in practical settings. For example, to model monthly counts, the model can be written as

Z_ti_ = β_0_ + β_C_ cos(2πωt_i_) + β_S_ sin(2πωt_i_) + ε_ti_ (3)

where, Z_ti_ is the count in the *t*th month of *i*th year; t values range from 1 to 12; *i* values range from 1 to L, where L is number of years under observation. In the context of the model ω reflects the period within every year, and for the monthly data, ω = 1/12. Thus, the above equation can be rewritten as

Z_ti_ = β_0_ + β_C_ cos(2πt_i_ /12) + β_S_ sin(2πt_i_/12) + ε_ti_. (4)

A linear combination of sine and cosine functions fits the seasonal variation in the outcome as a regular wave with a single, equally spaced peak and over the calendar year, with the actual position of the peak and trough guided by the data. The model parameters or beta coefficients β_0_, β_S_, β_C_ can be used to estimate peak timing and amplitude. 

The model can be extended to capture the trend of time series data, while adjusting for seasonality with the sine/cosine pair. For the example described above, including ‘*i*’ into the model can help to capture a long-term linear trend. Now, the harmonic regression model is rewritten as

Z_ti_ = β_0_ + β_C_ cos(2πt_i_/12) + β_S_ sin(2πt_i_/12) + β_Year_ i + ε_ti_ (5)

where, β_Year_ is the model parameter or beta coefficient of the trend variable. We adapted the above equation (5) for the Poisson-distributed outcome to form the base model

Model A: Y_ti_ = exp{β_0_ + β_C_ cos(2πt_i_/12) + β_S_ sin(2πt_i_/12) + β_Year_ i + ε_ti_} (6)

which we applied to our examples to fit monthly counts and estimate the peak time to enable the model extension. 

We estimated the peak timing, θ and the amplitude, α using the δ-method [17,25] using the following transformations: θ = M {arctan( β_S_ / β_C_ ) + k}/2π and α ={β_c_^2^ + β_s_^2^}^1/2^, where β_C_ and β_S_ were obtained from fitting Model A. The estimate of θ depends on join signs of β_C_ and β_S_; so *k* = 0, when both β_C_ and β_S_ are positive, *k* = 2π, when β_C_ < 0 and β_S_ > 0, and *k* = π, otherwise. Furthermore, standard deviations for amplitude α and peak timing θ can be also estimated.

### 2.2. Model Extensions

We extend the basic Model A to improve the fit by replacing cosine and sine functions with two wave functions: 2{1–cos(u)}/u^2^ and sin(u)/u, which are the Fourier transform/characteristic function of the symmetric triangular density and the uniform density, respectively. The advantage of using these functions is that their maximum value is 1 at a predefined time. Similarly to using a linear and quadratic terms in a simple regression model, the proposed cosine Fourier transform function 2{1–cos(u)}/u^2^ can be treated as a squared term of the sin(u/2)/(u/2). This quadratic form can be interpreted as acceleration to fit a sharper peak than the ordinary sine function. The derivation part is

2{1–cos(u)}/u^2^ = 2(2 sin^2^(u/2)/u^2^) = sin^2^ (u/2)/(u/2)^2^ = {sin(u/2)/(u/2)}^2^. (7)

Based on this property, we transform time t_i_ in Model A with u_i_ = 2πω(t_i_−θ) = 2π(t_i_−θ)/M, where θ is peak timing. For simplicity, the value θ can be estimated from the actual time series, as described in Equation (7) using the basic Model A.

Thus, Model B uses transformed time t_i_, as u_i_ = 2π(t_i_−θ)/12 and two wave functions

Model B: Y_ti_ = exp{β_0_ + β_C_ [2(1-cos u_i_)/u_i_^2^] + β_S_ [(sin u_i_)/u_i_] + β_Year_ i + ε_ti_}. (8)

Next, the basic Model A can be further extended to capture slight shifts in peak timing using simple transformations, such as

cos(u_i_ + 2πθ/12) = cos{(2π(t_i_−θ)/12) + (2πθ/12)} = cos{2πt_i/_12}

and

sin(u_i_ + 2πθ/12) = sin{(2π(t_i_−θ)/12) + (2πθ/12)} = sin{2πt_i/_12}.

These linear combinations of two wave functions form two additional Models C and D, respectively

Model C: Y_ti_ = exp{β_0_ + β_C_ [cos(u_i_ + 2πθ/12)] + β_S_ [(sin u_i_)/u_i_] + β_Year_ i + ε_ti_} (9)

and

Model D: Y_ti_ = exp{β_0_ + β_C_ [2 (1 - cos u_i_)/u_i_^2^] + β_S_ [sin(u_i_ + 2πθ/12)] + β_Year_ i + ε_ti_}. (10)

**Figure 1:**
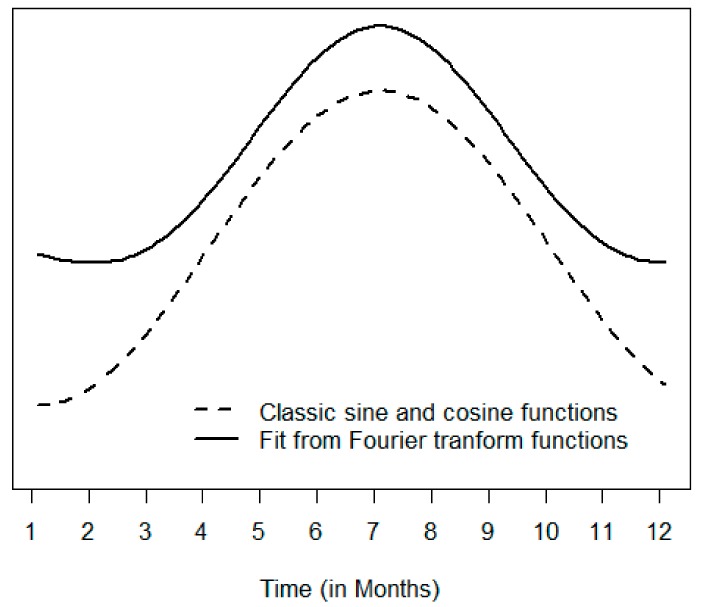
Smooth pattern of the classic sine and cosine functions (dashed line) and pattern of sine and cosine Fourier transform functions (solid line).

The proposed Models B, C, and D captures the variations in year, seasonal variation, and might be better tuned-up to capture the variations in seasonal amplitudes. The introduced terms based on Fourier transform functions can accommodate patterns with the sharp increase to reach the peak as shown in Figure 1. These models are likely to better describe the actual data.

### 2.3. Data

To illustrate the ability of the proposed models to capture trends and seasonal patterns we are using four examples based on the three actual datasets and one simulated dataset representing various infections occurred in specific populations. The datasets are presented in Supplemental Table S1. Below we provide a general description of infection’s etiology, epidemiology, and an applied data set. 

#### 2.3.1. Example 1: Hospitalizations Due to Salmonellosis in U.S. Elderly

Every year, *Salmonella* infection is estimated to cause over one million foodborne illnesses in the United States, with 19,000 hospitalizations and 380 deaths annually. Majority of infected with *Salmonella* develop diarrhea, fever, and abdominal cramps 12 to 72 hours after infection. The illness usually lasts 4 to 7 days, and most persons recover without treatment. In the frail elderly, however, the infection may be so severe that the patient needs to be hospitalized. The 25,367 hospital records of salmonellosis (ICD-9-CM 003.X) were extracted from the U.S. Centers of Medicare and Medicaid Services (CMS) database from 1991 to 2002. Each individual record contained age, admission date, and diagnosis codes [2]. In order to conduct time series analysis, records were organized as monthly counts observed among patients aged 65 and older. The aggregation of records into monthly time series of counts was based on patient admission date. 

#### 2.3.2. Example 2: Laboratory-Confirmed Cases of Shigellosis in Christian Medical College and Hospital, India

Shigellosis is an infectious disease caused by a group of bacteria called *Shigella* (shih-GEHL-uh) with a common fecal–oral transmission route via contaminated food or water. Most of the people who are infected with *Shigella* develop diarrhea, fever, abdominal pain, and dysentery (stools with blood and mucus) starting a day or two after they are exposed to the bacteria. Shigellosis usually resolves in 5 to 7 days. There may be asymptomatic carriers of the bacteria who are a source of infection to others. Effective and frequent handwashing, provision of safe drinking water and hygienic methods of food handling can stop transmission of shigellosis. The Department of Microbiology at CMC, Vellore receives stool samples that are sent for culture of common enteric pathogens. Stool samples of patients attending the emergency or outpatient departments or admitted to the hospital with a history of passing loose, frequent stools were collected and registered for culture. The diagnosis of shigellosis is made by successfully isolating the organism by conventional culture methods and identifying using specific antisera and appropriate biochemicals. 1242 records of positive cultures for *Shigella* were extracted from laboratory records between January 2003 and December 2013 and organized as monthly time series. 

#### 2.3.3. Example 3: Monthly Records of Pneumonia and Influenza Death in US

Influenza (flu) is a highly contagious viral infection which is one of the most severe illnesses of the winter season. Influenza is spread easily, when an infected person coughs or sneezes. Pneumonia is a serious infection or inflammation of the lungs, which can lead to death. Influenza is a common cause of pneumonia, especially among younger children, pregnant women, individuals with certain chronic health conditions, and frail elderly. While, in healthy individuals, flu rarely leads to pneumonia, those that do tend to be more severe and deadly. In fact, flu and pneumonia were the eighth leading cause of death in the United States in 2014. Monthly records of pneumonia and influenza death per 10,000 population in U.S. for 11 years between 1968 and 1978, representing 3,855 death events were abstracted from the public source [18]. The monthly rates were converted as per 1,000,000 population for computational convenience. 

#### 2.3.4. Example 4: Simulated Dataset 

Monthly counts for 132 time points were simulated based on Seasonal Auto Regressive Integrated Moving Average (SARIMA) model in R Version 3.3.2 (R Core Team, Vienna, Austria). This model involves six parameters, which are p (Auto Regression [AR]), d (Differencing [I]), q (Moving Average [MA]), P (Seasonal Auto Regression [SAR]), D (Seasonal Differencing [SI]), Q (Seasonal Moving Average [SMA]). The set of ((p, d, q), (P, D, Q), and S) defines the properties of a simulated sequence, where S is the time span of repeating seasonal pattern, thus for a monthly time series S = 12. To obtain a sequence of values with an increasing trend and apparent seasonality, the AR and SAR parameters are taken to be 0.6 and 0 respectively. The MA and SMA parameters control the past error, which are taken as 0.6 and 0 respectively; the I and SI parameters are taken as 0 and 1 respectively. Data were generated under a Poisson distribution assumption to simulate counts. 

## 3. Results

The inference based on case studies depends on size, prevalence, and seasonality of the specific diseases. Thus the case studies or data driven evidence of new model is unlikely to be robust. Therefore with the simulation we have introduced seasonality, trend using auto regression (AR) and moving average (MA) parameter values and the performance of the new model was compared with commonly used model.

In general the number of cases reported with Salmonelosis at the hospital has been declining. The rate of decline per year was by 7.4 counts, and Shigellosis infection is increasing over years at the rate of 0.6 counts per year. Flu data shows a declining trend with a rate of 0.9 counts per year. The dataset simulated showing an increasing trend with the rate of 7.8 counts per year.

The summary statistics for monthly values representing four examples are shown in Table 1. In addition to typical statistics, such as minimum, maximum, mean, standard deviations, first and third quartiles for monthly values and overall, we provide the estimates of coefficients of skewness and kurtosis (Table 2). 

Table 3 shows the results of root-mean-square error (RMSE), mean absolute deviance (MAD), Bayesian information criterion (BIC), and the regression coefficients for annual trend, sine and cosine terms for Models A, B, C, and D for four examples. Overall, all models provide relatively good fit to the data, yet the three applied statistics demonstrate potential model preference. While models are performing equally well in terms of RMSE and MAD, we consider BIC as a better measure for comparing between models.

Table 2 also contains the estimates of peak timing using the results of Model A. High values for skewness and kurtosis indicate the presence of sharp peaks in the studied time series of counts.

**Figure 2:**
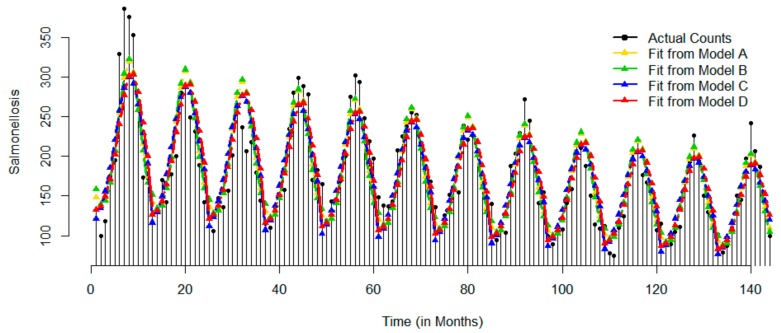
Time series of actual monthly records and superimposed with predicted values based on four models for salmonellosis: Models A–D are represented by yellow, green, blue, and red color, respectively.

Figure 2 shows the time series of monthly counts for salmonellosis for 12 years of the study period. Counts of salmonellosis have been decreasing from 1991 through 2002 showing a slow trend. The range of observed values decreased over time with more observations occurring within the range 131 (first Quartile) to 216 (third Quartile). Counts reached its maximum value of 386 in July 1991 and its minimum value of 75 in March 2000. The time series shows a clear seasonal patterns with high fluctuations in July (SD = 53.33) and low fluctuations in February (SD = 19.01) as compared to other months. Similarly, the peak (maximum count) occurs in August and the dip (minimum count) occurs in February. The results of Model A indicate that on average the counts peaked at 8.09 month, e.g., at the beginning of August. The time series plot of predicted values confirms a clear downward trend and a strong seasonal pattern, which account for substantial part of temporal variation, as evidenced by MAD. As shown in Table 3, Model B offers the best fit with RMSE (24.28) and BIC (1451.72) as compared to other models. 

**Figure 3:**
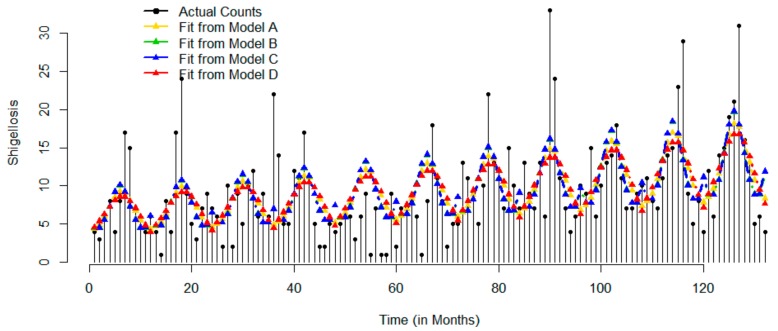
Time series of actual monthly records and superimposed with predicted values based on four models for shigellosis: Models A–D are represented by yellow, green, blue, and red color, respectively.

Figure 3 shows the time series of monthly counts for *Shigella*-related infections for 12-year study period. Counts of shigellosis have been slowly increasing from 2003 through 2013. The range of observed values increased over time with more observations ranging from 5 (first quartile) to 12 (third quartile) cases per month. Counts reached their maximum value of 33 in June 2010 and kept minimum value of 1 case in many months. The time series exhibit a clear seasonality with high fluctuations in June (SD = 8.47) and low fluctuations in November months (SD = 2.05). On average the peak occurs in June and the dip is in October. The results of Model A indicate that on average the counts peaked at 6.29 month, e.g., early-mid June. While visually the trend is not apparent and seasonality is hard to depict, all models had detected a modest but significant upward trend and significant seasonal component. As shown in Table 3 all models have overall low values for MAD. Model C had lowest RMSE (4.85) and BIC (842.96) values as compared to other models.

**Figure 4:**
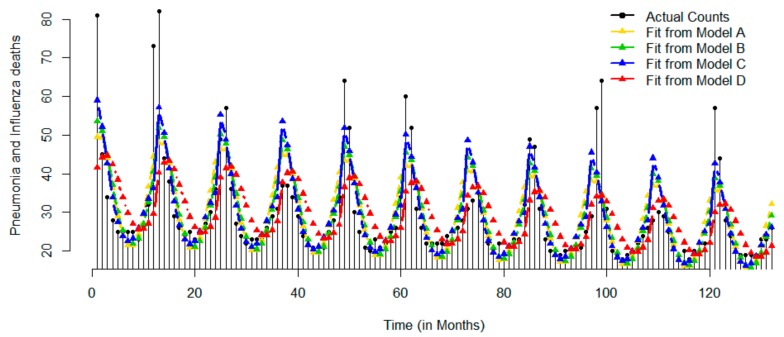
Time series of actual monthly records and superimposed with predicted values based on four models for pneumonia and influenza: Models A–D are represented by yellow, green, blue, and red color, respectively.

Figure 4 shows the time series of monthly death rates due to pneumonia and influenza for the 11-year period. Death rates showing a slow decreasing trend with the range of observed values ranging from 22 (first quartile) to 31 (third quartile) cases per month. Rates reached the maximum value of 82 cases in January of 1969 and the minimum value of 18 cases in July of 1976 and in June and August of 1977. The time series shows an obvious seasonality with high fluctuations in January (SD = 19.39) and low fluctuations in September (SD = 1.89). In general, the peak occurs in January and the dip was observed in August. The results of Model A indicate that on average death counts peaked at 1.47 month, e.g. mid-January. The time series plot of predicted values shows a downward trend and well-defined seasonal behavior, yet with somewhat irregular peaks, fluctuating between December and March. All models detected downward trend and seasonal patterns. Again, Model C had the best fit with lowest RMSE (7.11), MAD (4.15), and BIC (851.78) as compared to other models.

**Figure 5:**
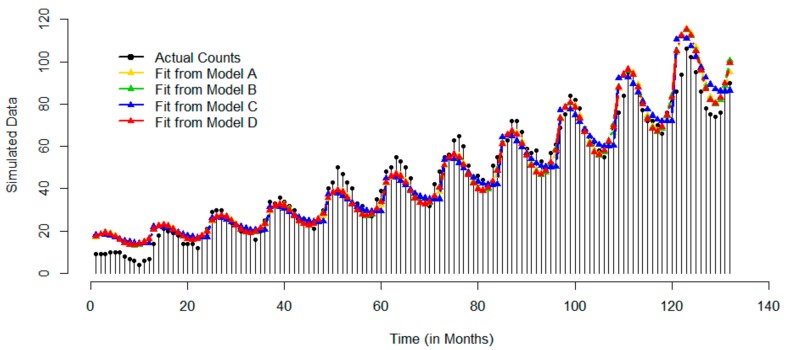
Time series of actual monthly records and superimposed with predicted values based on four models for simulated data: Models A–D are represented by yellow, green, blue, and red color, respectively.

Figure 5 shows that for simulated data, on average the peak (maximum count) occurs in March with the dip in October. Model A recovered the simulated peak at 3.21 months very well. The time series plot of the predicted values indicates that all models detected the strong upward trend and a significant seasonal component. Model B had a slight advantage over other models.

## 4. Discussion

To capture strong seasonal behavior with sharp peaks, we offered a two-step process when we first build a basic model and estimate the seasonal peak. We then apply an extended model using sine and cosine transform functions. These newly proposed functions mimic a quadratic term in the harmonic regression models and thus allow us to better fit the seasonal spikes. We illustrated the proposed method using actual and simulated data and can recommend the new approach to assess seasonality in a broad spectrum of diseases manifesting sharp seasonal peaks. 

In epidemiological and medical research, the regression methods are broadly used for the analysis of the time series data. The adaption of harmonic regression methodology is now well accepted to explore the trend and seasonality of diseases. Though three types of distributions: Gaussian, Poisson, and negative binomial assumed, most often Poisson harmonic regression is applied to accommodate the skewed nature of counts. The main drawback of harmonic regression is that it assumes a symmetrical nature of a harmonic process with the same rate of an increase and decrease in disease incidence from nadir to peak and vice versa [26]. Thus, by assuming a symmetric well-defined periodic structure, traditional harmonic models may not be ideal to capture the departures from stable oscillations [27]. The proposed approach could mitigate this problem.

There are difficulties involved in understanding and examining the concept of seasonality and its patterns as this need data to be collected over long time and over large spatial units. In the absence of such qualities, the evaluation may be affected by time-dependent and space-dependent confounders, which could possibly be improved by using a systematic approach to evaluation of seasonal curves [28], including parametric and non-parametric procedures of modeling [6], and non-linear methods developed with periodic functions in biology and climatology [25]. The proposed approach could further characterize disease seasonality. 

For example, the seasonal pattern of influenza infection is not completely understood due to the heterogeneity of infection transmission and manifestation. Few ideas of modeling complex influenza dynamics was explored, including the pyramid structure of disease burden with respect to severity of disease [14]. Wenger et al., analyzed 13 influenza seasons by developing methods to measure seasonality characteristics and to quantify the uncertainty of relevant parameters [5]. Wenger et al., also noted the seasonal peaks in varying heights which could be because of variation between individual years and detected a positive correlation between peak timing and amplitude, meaning that the early flu season arrival was typically high in intensity. While the uncertainty of seasonality parameters was assessed with delta-methods in [5,22], the bootstrap method was applied to find the confidence intervals [7]. In Eilers et al., to account for the varying annual seasonality in the disease counts, the coefficients of harmonics (sine and cosines) were allowed to vary smoothly over the age and time plane in the modelling in analyzing monthly deaths of respiratory diseases, in US female for years 1958–1998 and for ages 51–100. The over-dispersion encountered during tuning the smoothness parameter, handled with selective weighting and by using quasi-likelihood instead of Poisson [29]. Negative binomial regression model with harmonic terms could be preferred over Poisson model due to the overdispersion confirmed by a statistical test. Chui et al., introduced graphical tools, so-called multi-panel graphs, to visualize simultaneously the population structure and temporal trend and to link the graphs to models like harmonic regression to ease the interpretation of models results. This graphical approach was applied for four datasets: Influenza and Salmonella associated hospitalizations, confirmed salmonellosis cases, asthma-related hospital visits in USA [30]. The spatiotemporal patterns of influenza associated hospitalizations were also analyzed using harmonic regression with an additional squared time term to account for a quadratic trend component along with the linear trend component [31]. The limitation of this study was that the simulation should have been done with various sample sizes and means. However, this study indicated that harmonic regression was meant for sharp peaks based on corroborative evidences from the case studies

In the proposed study, we had illustrated that by adapting a two-step approach to commonly accepted models that can be easily implemented with existing statistical open-source software, the fit of the models can be further improved. The proposed approach can be broadly adapted to a wide range of scenarios when researchers are looking to statistical tools to formally compare the time series in different populations or across different time periods. In comparing time series, characterization of trend and seasonality are the key components of the analysis. For example, the outcome of interest might be the time series of disease incidence in a specific location and the task is to determine whether there is an overall decline in incidence in presence of strong seasonal variations with a likely complex form. In fact, we know that monthly cholera occurrences observed over 11 years exhibit a decreasing trend and a strong seasonality with high incidence in July and August [4,32,33,34,35,36]. Our method would allow to find a more refined fit to existing data and offer better interpretation of the obtained results as compared to the traditional approaches.

The suggested two-step approach can be further improved by exploring harmonic terms with additional sine/cosine pairs at shorter or longer wavelengths, which should be able to accommodate a more complex temporal behavior. The typical periodic oscillations well defined in epidemiology occurred on a weekly, monthly, or quarterly basis and therefore these cycles are observed within a year. We recognize the limitation in using monthly values, which offer a coarser estimate than could be offered by refined time units, like days or weeks [26]. In order to improve the descriptive power of the regression models adapted to time series of counts to detect these cycles, it would be valuable to examine how the degree of temporal aggregation affect the accuracy and precision. It is likely that the proposed two-step procedure will improve with increasing temporal resolution, e.g., by replacing monthly counts with weekly or daily time series of counts. In our study, we use counts as an outcome without adjusting for population (except for pneumonia and flu deaths rates). We assumed the population is unlikely to meaningfully change over the study periods and affect the seasonality estimates. Further studies could explore the factors that affect trends, including changes in exposed population.

The suggested two-step approach provides a solution for fitting sharp peaks with simple transforms when peak timing is unknown. In general, peak timing can be roughly estimated by superimposing monthly counts over years of the time series data for the study period. One can also assume a discrete probability model for θ, so the probability masses can be estimated as the frequency ratios of θ_t_, the corresponding model would represent a harmonic mixture regression model.

Another direction for further adaptation of the proposed model is to expand the model by allowing additional factors that may help to explain trend and seasonal variation or account for confounding. As we limited our focus in introducing a model building strategy with new Fourier terms, we did not explore potential exposure variables. That could possibly be explored further. Thus, the interest of a study might be to identify factors that influenced a trend of a disease. Such factors could be represented by environmental, clinical, or demographic variables [7,8,9,10,26,27,31,37,38]. For example, in investigating the factors associated with seasonal peaks of cholera various environmental, climatic, and meteorological factors demonstrated potential links and provide an insight to underlying mechanisms [4]. We also assumed that the strong autocorrelation in the outcome time series is not of major concern after controlling for seasonality and trend patterns [39]. Yet, such an assumption should be further tested along with the assumption of a true annual period [26].

Applications of time series analysis is gaining momentum and the growing interest of public health professionals, epidemiologists, and clinicians for a better understanding and quantification of temporal variations in disease incidence require proper tools to conduct such research with increased accuracy.

## 5. Conclusions

Though numerous well proved complex models are available for time series data analysis, researchers prefer and use regression-based methods because of their diversity and flexibility in adopting amendments during model building procedures. While using these methods for convenience, unintentionally many inherent qualities of time series data are neglected in modeling. We already have well accepted harmonic regression model that provide good fit for trend and stable periodic patterns with relatively symmetric rises and falls. The new proposed model is compared and evaluated with the existing model using real time and simulated datasets, based on the fit statistics and other values. The newly developed model can handle time series data with sharp sporadic peaks and prolong periods of low incidence and could offer an advantage over the traditional harmonic regression model. 

## Figures and Tables

**Table 1:** Summary statistics for four examples: salmonellosis, shigellosis, pneumonia and influenza and simulated monthly counts for overall time period and by the month of the study period

Statistics	Jan	Feb	Mar	Apr	May	Jun	Jul	Aug	Sep	Oct	Nov	Dec	Overall
**Example 1: Salmonellosis**													
Mean	129.3	103.5	120.5	131.1	161.3	198.5	249.8	262.5	239.9	209.7	163.6	144.4	176.2
SD	20.4	19.0	28.2	23.0	28.3	50.1	53.3	48.0	52.0	42.7	29.1	31.5	63.5
Min	99	78	75	104	111	145	193	211	176	150	114	100	75
Max	165	138	170	175	208	329	386	376	353	278	219	197	386
1st Qrt	113.5	89.5	96.5	109	145.5	161.5	209	223.5	200	177	142	116.5	131
3rd Qrt	143.5	114.5	140	145.5	182.5	214	277.5	294.5	270.5	239.5	183.5	170	216
**Example 2: Shigellosis**													
Mean	8.9	6.6	7.8	8.1	10.0	16.0	15.9	12.2	6.4	5.7	7.0	7.4	9.3
SD	3.9	4.1	2.5	4.4	5.5	8.5	8.3	6.6	3.4	3.9	2.1	5.5	6.1
Min	4	1	5	2	1	5	1	5	1	1	4	2	1
Max	14	15	14	15	19	33	31	29	13	15	10	22	33
1st Qrt	5	3	6	4	6	8	10	7	3	2	5	4	5
3rd Qrt	13	9	9	12	14	22	23	15	8	7	9	10	12
**Example 3: Pneumonia and Influenza**													
Mean	51.6	45.3	35.5	27.1	22.5	21.0	21.6	20.9	21.2	24.1	25.8	34.1	29.2
SD	19.4	9.1	10.0	2.7	2.0	2.3	2.4	2.2	1.9	2.0	3.6	13.4	12.6
Min	28	30	28	23	20	18	18	18	19	21	21	25	18
Max	82	57	64	31	26	25	25	25	24	27	32	73	82
1st Qrt	31	37	30	25	21	19	19	19	19	22	23	26	22
3rd Qrt	64	52	36	29	24	23	23	23	23	26	29	34	31
**Example 4: Simulated Data**													
Mean	47.5	50.5	56.5	55.1	51.3	46.2	41.3	40.8	38.6	36.4	43.6	47.7	46.3
SD	24.9	27.1	31.1	30.6	24.4	24.9	23.9	23.4	22.9	23.2	25.8	26.1	25.8
Min	9	9	9	10	10	10	8	7	6	4	6	7	4
Max	86	94	106	102	95	86	78	75	74	76	88	90	106
1st Qrt	29	30	30	27	24	23	20	21	20	16	20	25	24.5
3rd Qrt	69	75	84	82	78	69	64	62	58	55	62	68	66.5

**Table 2:** Summary statistics and seasonality characteristics for four examples: salmonellosis, shigellosis, pneumonia, and influenza and simulated monthly counts for overall time period

Statistics	Example 1: Salmonellosis	Example 2: Shigellosis	Example 3: Pneumonia	Example 4: Simulated Data
Skewness (SE)	0.85 (0.20)	1.44 (0.21)	2.23 (0.21)	0.25 (0.21)
Kurtosis (SE)	0.58 (0.40)	2.63 (0.42)	5.17 (0.42)	−0.93 (0.42)
Peak timing (SE)	8.09 (0.07)	6.29 (0.47)	1.47 (0.12)	3.21 (0.26)
Amplitude (SE)	0.42 (0.02)	0.39 (0.08)	0.43 (0.02)	0.18 (0.03)

**Table 3:** Comparison of models for four examples: salmonellosis, shigellosis, pneumonia, and influenza and simulated monthly counts

Estimator	Model A	Model B	Model C	Model D
**Example 1: Salmonellosis**				
Constant	5.39 (5.37 to 5.42; < 0.001)	7.07 (6.79 to 7.35; < 0.001)	4.93 (4.90 to 4.97; < 0.001)	4.79 (4.71 to 4.87; < 0.001)
Year	−0.04 (−0.05 to −0.04; < 0.001)	−0.04 (−0.05 to −0.04; < 0.001)	−0.04 (−0.05 to −0.04 < 0.001)	−0.04 (−0.05 to −0.04; < 0.001)
Sin	−0.37 (−0.39 to −0.36; < 0.001)	3.91 (3.50 to 4.32; < 0.001)	0.82 (0.78 to 0.86; < 0.001)	−0.18 (−0.21 to −0.15; < 0.001)
Cos	−0.19 (−0.21 to −0.17; < 0.001)	−5.16 (−5.84 to −4.48; < 0.001)	0.02 (0.00 to 0.04; 0.047)	0.80 (0.70 to 0.90; < 0.001)
RMSE	24.47	24.28	28.58	29
MAD	18.76	18.66	22.67	22.41
BIC	1458.28	1451.72	1664.47	1643.73
**Example 2: Shigellosis**				
Constant	1.77 (1.63 to 1.90; < 0.001)	7.92 (5.63 to 10.17; < 0.001)	3.28 (2.60 to 3.95; < 0.001)	0.76 (0.46 to 1.04; < 0.001)
Year	0.07 (0.05 to 0.09; < 0.001)	0.07 (0.05 to 0.09; < 0.001)	0.07 (0.05 to 0.09; < 0.001)	0.07 (0.05 to 0.09; < 0.001)
Sin	−0.06 (−0.14 to 0.02; 0.140)	9.58 (6.52 to 12.60; < 0.001)	−2.58 (−3.71 to −1.43; < 0.001)	−0.06 (−0.14 to 0.02; 0.146)
Cos	−0.39 (−0.47 to −0.31; < 0.001)	−15.26 (−20.5 to −9.97; < 0.001)	−1.55 (−2.06 to −1.02; < 0.001)	1.32 (1.01 to 1.64; < 0.001)
RMSE	4.93	4.86	4.85	5.09
MAD	3.59	3.53	3.52	3.68
BIC	859.66	844.31	842.96	878.93
**Example 3: Pneumonia and Influenza**				
Constant	3.52 (3.45 to 3.58; < 0.001)	3.92 (3.81 to 4.03; < 0.001)	3.39 (3.32 to 3.46; < 0.001)	3.42 (3.31 to 3.52; < 0.001)
Year	−0.03 (−0.04 to −0.02; < 0.001)	−0.03 (−0.04 to −0.02; < 0.001)	−0.03 (−0.04 to −0.02; < 0.001)	−0.03 (−0.04 to −0.02; < 0.001)
Sin	0.30 (0.25 to 0.35; < 0.001)	2.00 (1.73 to 2.27; < 0.001)	0.48 (0.42 to 0.55; < 0.001)	0.18 (0.10 to 0.27; < 0.001)
Cos	0.31 (0.26 to 0.35; < 0.001)	−1.91 (−2.24 to −1.57; < 0.001)	0.28 (0.23 to 0.32; < 0.001)	0.25 (0.08 to 0.42; 0.004)
RMSE	7.96	7.49	7.11	10.37
MAD	5.22	4.74	4.15	6.97
BIC	896.19	870.76	851.78	1064.65
**Example 4: Simulated Data**				
Constant	2.59 (2.52 to 2.66; < 0.001)	3.06 (2.86 to 3.26; < 0.001)	2.49 (2.41 to 2.56; < 0.001)	2.63 (2.52 to 2.73; < 0.001)
Year	0.18 (0.17 to 0.19; < 0.001)	0.18 (0.17 to 0.19; < 0.001)	0.18 (0.17 to 0.19; < 0.001)	0.18 (0.17 to 0.19; < 0.001)
Sin	0.18 (0.14 to 0.21; < 0.001)	1.33 (0.96 to 1.70; < 0.001)	0.24 (0.18 to 0.30; < 0.001)	0.20 (0.14 to 0.25; < 0.001)
Cos	−0.02 (−0.05 to 0.02; 0.396)	−1.63 (−2.16 to −1.09; < 0.001)	0.04 (0.01 to 0.08; 0.026)	−0.05 (−0.17 to 0.07; 0.383)
RMSE	5.79	5.79	6.76	5.79
MAD	4.62	4.62	5.27	4.63
BIC	870.4	869.87	900.36	870.36

Root-mean-square error (RMSE); mean absolute deviance (MAD); Bayesian information criterion (BIC).

## Supplementary Materials

Table S1: Monthly counts of salmonellosis, shigellosis, pneumonia, and influenza infections and simulated data. The following are available online at https://www.mdpi.com/1660-4601/17/4/1318/s1.
