# Supplementary material for: Assessing Seasonality Variation with Harmonic Regression: Accommodations for Sharp Peaks
Source: Int J Environ Res Public Health. 2020 Feb 18;17(4):1318. doi: 10.3390/ijerph17041318 (PMC7068504; doi:10.3390/ijerph17041318)
Supplement: Supplementary file 1 [file ijerph-17-01318-s001.pdf]

Table S1: Monthly counts of Salmonellosis, Shigellosis, Pneumonia and Influenza infections and Simulated data

Salmonellosis			Shigellosis			Pneumonia and Influenza			Simulated Data		
Year	Month	Count	Year	Month	Count	Year	Month	Count	Year	Month	Count
1991	1	133	2003	1	4	1968	1	81	1	1	9
1991	2	99	2003	2	3	1968	2	45	1	2	9
1991	3	118	2003	3	6	1968	3	34	1	3	9
1991	4	175	2003	4	8	1968	4	28	1	4	10
1991	5	195	2003	5	4	1968	5	25	1	5	10
1991	6	329	2003	6	8	1968	6	25	1	6	10
1991	7	386	2003	7	17	1968	7	25	1	7	8
1991	8	376	2003	8	15	1968	8	25	1	8	7
1991	9	353	2003	9	7	1968	9	23	1	9	6
1991	10	270	2003	10	6	1968	10	26	1	10	4
1991	11	173	2003	11	4	1968	11	32	1	11	6
1991	12	172	2003	12	6	1968	12	73	1	12	7
1992	1	142	2004	1	4	1969	1	82	2	1	14
1992	2	130	2004	2	1	1969	2	44	2	2	18
1992	3	170	2004	3	8	1969	3	38	2	3	21
1992	4	142	2004	4	4	1969	4	29	2	4	20
1992	5	177	2004	5	17	1969	5	26	2	5	19
1992	6	200	2004	6	24	1969	6	24	2	6	18
1992	7	280	2004	7	9	1969	7	25	2	7	14
1992	8	290	2004	8	5	1969	8	23	2	8	14
1992	9	249	2004	9	3	1969	9	23	2	9	14
1992	10	231	2004	10	7	1969	10	27	2	10	12
1992	11	189	2004	11	9	1969	11	30	2	11	17
1992	12	142	2004	12	7	1969	12	36	2	12	21
1993	1	145	2005	1	6	1970	1	49	3	1	29
1993	2	106	2005	2	2	1970	2	57	3	2	30
1993	3	147	2005	3	10	1970	3	36	3	3	30
1993	4	136	2005	4	2	1970	4	27	3	4	27
1993	5	157	2005	5	9	1970	5	24	3	5	24
1993	6	201	2005	6	5	1970	6	22	3	6	23
1993	7	263	2005	7	10	1970	7	23	3	7	20
1993	8	237	2005	8	12	1970	8	23	3	8	21
1993	9	207	2005	9	6	1970	9	23	3	9	20
1993	10	218	2005	10	9	1970	10	26	3	10	16
1993	11	179	2005	11	6	1970	11	29	3	11	20
1993	12	144	2005	12	22	1970	12	31	3	12	25
1994	1	115	2006	1	14	1971	1	37	4	1	34
1994	2	110	2006	2	5	1971	2	37	4	2	33
1994	3	136	2006	3	5	1971	3	34	4	3	36
1994	4	148	2006	4	12	1971	4	29	4	4	34
1994	5	158	2006	5	11	1971	5	24	4	5	32
1994	6	235	2006	6	17	1971	6	23	4	6	30

1994	7	280	2006	7	11	1971	7	21	4	7	26
1994	8	299	2006	8	5	1971	8	21	4	8	26
1994	9	289	2006	9	2	1971	9	21	4	9	23
1994	10	278	2006	10	2	1971	10	25	4	10	21
1994	11	170	2006	11	5	1971	11	28	4	11	26
1994	12	183	2006	12	4	1971	12	34	4	12	30
1995	1	165	2007	1	5	1972	1	64	5	1	40
1995	2	119	2007	2	6	1972	2	52	5	2	43
1995	3	143	2007	3	6	1972	3	30	5	3	50
1995	4	141	2007	4	3	1972	4	25	5	4	47
1995	5	169	2007	5	6	1972	5	21	5	5	43
1995	6	200	2007	6	9	1972	6	20	5	6	40
1995	7	275	2007	7	0	1972	7	23	5	7	33
1995	8	302	2007	8	7	1972	8	21	5	8	32
1995	9	294	2007	9	1	1972	9	22	5	9	29
1995	10	248	2007	10	1	1972	10	25	5	10	27
1995	11	219	2007	11	9	1972	11	26	5	11	35
1995	12	197	2007	12	2	1972	12	34	5	12	39
1996	1	148	2008	1	7	1973	1	60	6	1	48
1996	2	138	2008	2	7	1973	2	52	6	2	50
1996	3	137	2008	3	8	1973	3	31	6	3	55
1996	4	143	2008	4	6	1973	4	26	6	4	53
1996	5	208	2008	5	1	1973	5	22	6	5	50
1996	6	225	2008	6	8	1973	6	22	6	6	45
1996	7	238	2008	7	18	1973	7	22	6	7	37
1996	8	255	2008	8	11	1973	8	22	6	8	36
1996	9	252	2008	9	8	1973	9	24	6	9	33
1996	10	217	2008	10	2	1973	10	25	6	10	32
1996	11	188	2008	11	5	1973	11	26	6	11	42
1996	12	168	2008	12	5	1973	12	28	6	12	48
1997	1	136	2009	1	13	1974	1	31	7	1	55
1997	2	110	2009	2	11	1974	2	33	7	2	56
1997	3	126	2009	3	9	1974	3	35	7	3	63
1997	4	151	2009	4	5	1974	4	31	7	4	65
1997	5	158	2009	5	10	1974	5	22	7	5	60
1997	6	155	2009	6	22	1974	6	20	7	6	51
1997	7	239	2009	7	13	1974	7	22	7	7	45
1997	8	221	2009	8	12	1974	8	19	7	8	46
1997	9	226	2009	9	7	1974	9	20	7	9	44
1997	10	203	2009	10	15	1974	10	23	7	10	42
1997	11	163	2009	11	10	1974	11	23	7	11	51
1997	12	132	2009	12	7	1974	12	32	7	12	55
1998	1	140	2010	1	13	1975	1	49	8	1	62
1998	2	94	2010	2	9	1975	2	47	8	2	63
1998	3	111	2010	3	7	1975	3	31	8	3	72
1998	4	104	2010	4	13	1975	4	23	8	4	72
1998	5	188	2010	5	6	1975	5	20	8	5	67

1998	6	203	2010	6	33	1975	6	19	8	6	59
1998	7	229	2010	7	24	1975	7	19	8	7	57
1998	8	272	2010	8	11	1975	8	20	8	8	58
1998	9	245	2010	9	7	1975	9	19	8	9	53
1998	10	197	2010	10	4	1975	10	21	8	10	49
1998	11	141	2010	11	6	1975	11	21	8	11	57
1998	12	155	2010	12	10	1975	12	25	8	12	61
1999	1	99	2011	1	9	1976	1	29	9	1	69
1999	2	89	2011	2	15	1976	2	57	9	2	75
1999	3	104	2011	3	6	1976	3	64	9	3	84
1999	4	108	2011	4	10	1976	4	31	9	4	82
1999	5	141	2011	5	13	1976	5	20	9	5	78
1999	6	159	2011	6	14	1976	6	19	9	6	69
1999	7	215	2011	7	18	1976	7	18	9	7	64
1999	8	211	2011	8	11	1976	8	19	9	8	62
1999	9	188	2011	9	7	1976	9	19	9	9	58
1999	10	150	2011	10	7	1976	10	22	9	10	55
1999	11	114	2011	11	9	1976	11	24	9	11	62
1999	12	109	2011	12	10	1976	12	26	9	12	68
2000	1	112	2012	1	11	1977	1	28	10	1	76
2000	2	78	2012	2	8	1977	2	30	10	2	84
2000	3	75	2012	3	7	1977	3	29	10	3	95
2000	4	110	2012	4	11	1977	4	25	10	4	94
2000	5	124	2012	5	14	1977	5	21	10	5	86
2000	6	164	2012	6	15	1977	6	18	10	6	77
2000	7	203	2012	7	23	1977	7	20	10	7	72
2000	8	219	2012	8	29	1977	8	18	10	8	72
2000	9	176	2012	9	9	1977	9	20	10	9	70
2000	10	167	2012	10	5	1977	10	22	10	10	66
2000	11	154	2012	11	8	1977	11	22	10	11	76
2000	12	107	2012	12	4	1977	12	30	10	12	81
2001	1	115	2013	1	12	1978	1	57	11	1	86
2001	2	90	2013	2	6	1978	2	44	11	2	94
2001	3	89	2013	3	14	1978	3	28	11	3	106
2001	4	105	2013	4	15	1978	4	24	11	4	102
2001	5	111	2013	5	19	1978	5	22	11	5	95
2001	6	166	2013	6	21	1978	6	19	11	6	86
2001	7	193	2013	7	31	1978	7	19	11	7	78
2001	8	226	2013	8	16	1978	8	19	11	8	75
2001	9	194	2013	9	13	1978	9	19	11	9	74
2001	10	150	2013	10	5	1978	10	23	11	10	76
2001	11	130	2013	11	6	1978	11	23	11	11	88
2001	12	124	2013	12	4	1978	12	26	11	12	90
2002	1	102									
2002	2	79									
2002	3	87									
2002	4	110									

2002	5	150
2002	6	145
2002	7	197
2002	8	242
2002	9	206
2002	10	187
2002	11	143
2002	12	100
